# Comprehensive Review of Nystagmus and Vertigo Diagnostics: From Pathological Foundations to AI-Driven Telemedicine

**DOI:** 10.3390/s26123949

**Published:** 2026-06-22

**Authors:** Kowshik Balasubramanian, Ali Danesh, Abhijit Pandya

**Affiliations:** 1 Department of Electrical Engineering and Computer Science, College of Engineering and Computer Science, Florida Atlantic University, Boca Raton, FL 33431, USA; 2 Hearing, Tinnitus, Balance Research Laboratory, Department of Communication Sciences and Disorders, Schmidt College of Medicine, Florida Atlantic University, Boca Raton, FL 33431, USA; 3 Department of Biomedical Sciences, Schmidt College of Medicine, Florida Atlantic University, Boca Raton, FL 33431, USA; 4 Department of Biomedical Engineering, College of Engineering and Computer Science, Florida Atlantic University, Boca Raton, FL 33431, USA

**Keywords:** nystagmus, vertigo, BPPV, vestibular diagnostics, deep learning, machine learning, smartphone oculography, telemedicine, slow-phase velocity, video-oculography

## Abstract

Nystagmus, the involuntary rhythmic oscillation of the eyes, is a critical diagnostic marker in vestibular medicine, distinguishing life-threatening central disorders such as stroke from benign peripheral conditions including Benign Paroxysmal Positional Vertigo (BPPV). Despite its clinical importance, accurate nystagmus assessment has long been constrained by expensive infrared video-oculography equipment such as videonystagmography, specialist dependency, and the episodic nature of vestibular symptoms that are often resolved before a clinical encounter. This review synthesizes approximately 50 papers published between 1952 and 2026 across four thematic domains: AI-driven nystagmus analysis, clinical medicine, smartphone and portable hardware innovations, and telemedicine and remote monitoring. On the AI front, classical machine learning models achieve up to 98.77% nystagmus recognition accuracy using ensemble methods, while deep learning frameworks spanning CNNs, U-Nets, LSTMs, and optical flow networks demonstrate clinical-grade slow-phase velocity measurement equivalent to gold standard video-oculography on standard smartphone RGB video. Large language and vision models including GPT-4V and Gemini 2.0 show early-stage promise as zero-shot triage tools but currently fall well below specialist-level diagnostic accuracy. Concurrently, portable hardware innovations ranging from 3D-printed goggle systems to ARKit-based smartphone applications are narrowing the accessibility gap, while telemedicine frameworks enable ictal recording and cloud-based specialist review outside the clinic. Across all domains, the common barriers to clinical translation are dataset scarcity for rare BPPV subtypes, sensitivity to ambient conditions, and the absence of explainable AI mechanisms. This review maps the current state of the field and identifies multimodal data fusion, prospective clinical validation, and interpretable AI as the critical next steps toward equitable, specialist independent vestibular diagnostics.

## 1. Introduction

Dizziness and vertigo are among the most prevalent and diagnostically challenging complaints in clinical medicine, with a lifetime prevalence of vestibular vertigo estimated at 7.4% in the general population and a one-year prevalence of 4.9% [1]. In the United States alone, dizziness accounts for approximately 4% of all emergency department (ED) chief complaints, generating an estimated 3.9 million ED visits annually and exceeding USD 4 billion in direct costs, a figure driven substantially by overreliance on low yield neuroimaging [2]. Despite this volume, diagnostic accuracy remains poor: stroke, the most dangerous cause of acute dizziness, is missed in the ED approximately 17% of the time overall, and patients presenting with isolated dizziness or vertigo without focal neurological deficits face misdiagnosis rates approaching 35–40% [3]. The consequences of missed central diagnoses, particularly posterior circulation strokes and cerebellar infarction, are severe, including brainstem compression, hydrocephalus, and preventable death [4]. The scale and consequence of this diagnostic failure establish vestibular medicine as a domain where systematic, objective, and accessible tools are not merely desirable but clinically necessary.

The spectrum of vestibular disorders encompasses both benign self-limiting conditions and neurological emergencies that demand immediate intervention. The landmark pathological work of Dix and Hallpike in 1952 established the foundational clinical distinctions between Meniere’s disease, vestibular neuronitis, and BPPV, and introduced the positional maneuver that remains the diagnostic gold standard for posterior canal BPPV [5]. Peripheral disorders including Benign Paroxysmal Positional Vertigo (BPPV), vestibular neuritis, and Meniere’s disease account for most vestibular presentations and are generally benign, episodic, and treatable with repositioning maneuvers or conservative management [6]. Central disorders, particularly posterior circulation stroke, can present identical symptoms of acute dizziness, nausea, and gait instability, yet carry fundamentally different prognoses requiring emergency intervention [7,8]. The critical clinical task distinguishing peripheral from central etiologies at first presentation requires the assessment of nystagmus characteristics that are subtle, transient, and interpretable only by trained specialists, creating a structural gap in diagnostic capacity that disproportionately affects emergency and primary care settings.

The current gold standard for objective nystagmus assessment is infrared video-oculography (VOG) also known as videonystagmography VNG, capable of quantifying slow-phase velocity, direction, and waveform morphology with high fidelity. However, clinical-grade VOG systems cost up to USD 40,000, require trained technicians to operate, and are confined to subspecialty vestibular clinics [9]. Emergency departments and primary care settings where the majority of dizzy patients first present are left dependent on bedside clinical examination, which is subject to significant interobserver variability and requires training that most non-specialist clinicians do not receive [8]. Compounding this, vestibular symptoms are episodic by nature: BPPV resolves between attacks, Meniere’s disease manifests in discrete crises, and the window for capturing diagnostic nystagmus is often closed by the time a patient reaches a specialist. These structural barriers’ cost, expertise, and timing create the diagnostic gap that the technologies reviewed in this paper are collectively attempting to close [10,11].

The past decade has produced a rapid convergence of artificial intelligence, smartphone hardware, and telemedicine infrastructure that is fundamentally reshaping what objective vestibular assessment can look like outside the specialist clinic. Convolutional neural networks fine-tuned on facial and pupil landmarks now produce slow-phase velocity measurements statistically equivalent to gold-standard VOG using standard 30 Hz RGB smartphone video [12]. Machine learning models integrating multimodal clinical features outperform established bedside scores such as HINTS and ABCD2 in differentiating central from peripheral etiologies [13]. Patient-initiated home recording systems capture diagnostic nystagmus during active attacks data that would otherwise be invisible to the clinical record [14]. Systematic reviews of this rapidly expanding literature confirm both the clinical promise and the persistent methodological limitations: dataset heterogeneity, lack of prospective validation, and the absence of explainable AI mechanisms that would support regulatory approval and clinician trust [15].

This review synthesizes approximately 50 papers published between 1952 and 2026, organized into four thematic sections: AI-driven nystagmus analysis (machine learning, deep learning, and large language models), medicine-based clinical studies, smartphone and portable hardware innovations, and telemedicine and remote monitoring frameworks. The literature review documents the primary contributions, methods, and findings of each included work. A dedicated analysis section connects findings across papers within each domain, identifying convergent trends, unresolved tensions, and the most consequential open problems for the field. The aim is to provide researchers and clinicians with a structured map of where the current technology stands, where it falls short, and what is required to bring specialist-grade vestibular diagnostics to the point of care.

## 2. Literature Review

**Figure 1:**
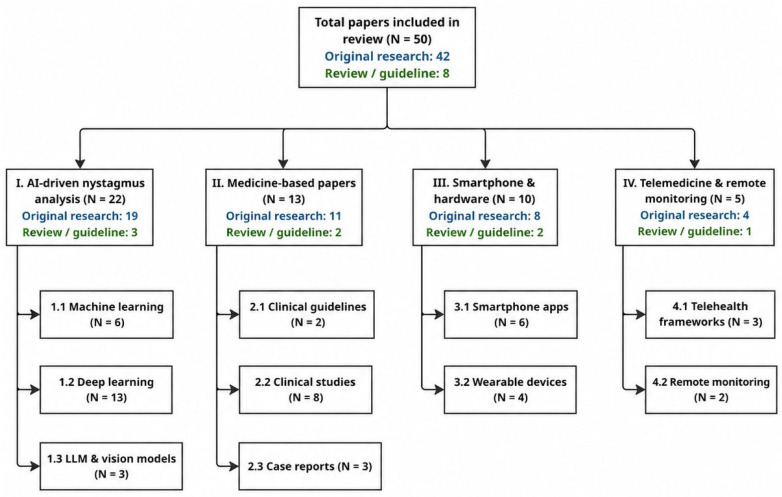
Hierarchical overview of the review structure, showing the four thematic sections, their subsections, and the number of papers in each category. Papers are further classified as original research or review/guideline works.

The literature for this review was identified through structured searches of PubMed, IEEE Xplore, Scopus, and Google Scholar, supplemented by manual screening of the reference lists of relevant articles. Search terms combined vestibular and oculomotor concepts with computational and hardware terms, including ‘nystagmus’, ‘vertigo’, ‘BPPV’, ‘video-oculography’, ‘videonystagmography’, ‘slow-phase velocity’, ‘machine learning’, ‘deep learning’, ‘smartphone’, and ‘telemedicine’, in various combinations. Studies were included if they addressed objective nystagmus or vestibular assessment through AI-driven analysis, clinical diagnostic methodology, portable or smartphone-based hardware, or telemedicine and remote monitoring, and were excluded if they did not report a diagnostic or technical method relevant to nystagmus or vestibular evaluation. Because this review spans heterogeneous study designs from foundational pathology and clinical guidelines to engineering proof-of-concept frameworks, a formal quality-appraisal instrument was not applied; instead, each work was assessed qualitatively for methodological transparency, validation design, and relevance to clinical translation. This process yielded approximately 50 papers published between 1952 and 2026, with the 1952 lower bound chosen to capture the foundational clinicopathological work of Dix and Hallpike and the majority concentrated in 2019–2026, reflecting the rapid recent growth of Artificial Intelligence (AI)- and smartphone-based diagnostic tools in vestibular medicine. Of the 50 papers, 8 are classified as review or guideline works comprising 3 systematic reviews, 1 meta-analysis, 2 narrative or literature surveys, and 2 evidence-based clinical guidelines while the remaining 42 are original research contributions encompassing prospective studies, retrospective analyses, pilot studies, case reports, and proof-of-concept frameworks. The papers span multiple disciplines including neuro-otology, emergency medicine, computer vision, signal processing, and machine learning. Methodologically, the corpus covers a wide range of diagnostic modalities: infrared and visible-light video-oculography, smartphone-based eye tracking, videonystagmography, video head-impulse (vHIT) testing, augmented reality headsets, wearable sensors, and 3D-printed hardware. On the computational side, approaches range from classical machine learning algorithms including support vector machines, random forests, and gradient boosting to deep learning architectures such as Convolutional Neural Networks (CNN), U-Nets, Long Short-Term Memory (LSTM), and optical flow networks, as well as large language and vision models including GPT-4V and Gemini 2.0. The reviewed literature is organized into four thematic sections, as illustrated in Figure 1.

### 2.1. Artificial Intelligence in Vestibular Diagnostics: From Classical Models to Foundation Models

#### 2.1.1. Classical Machine Learning for Vestibular Disease Classification and Triage

Lai et al. address the critical diagnostic challenge of distinguishing dangerous vertigo (central or life-threatening conditions like stroke) from BPPV (peripheral or self-limiting disorders) in outpatient settings [16]. Their research is particularly notable for addressing diagnostic heterogeneity by validating a model across two distinct medical institutions that utilize different video-oculography (VOG) and video head impulse test (vHIT) equipment and protocols. The proposed framework utilizes an optimized Random Forest (RF) classifier integrated with Adaptive Boosting (AdaBoost) to process 24 shared features, including patient demographics and ocular metrics from fixation, gaze, smooth pursuit, and saccade tests.

Ahmadi et al. address the critical diagnostic challenge of differentiating between central (vestibular stroke) and peripheral (vestibular neuritis) etiologies in patients presenting with acute vestibular symptoms [13]. The study utilized a multimodal dataset from 108 emergency department patients, incorporating standardized neuro-otological examinations, quantitative video-oculography (VOG), and MRI findings. The researchers benchmarked established clinical scores, such as HINTS and ABCD2, against several machine-learning architectures, including linear logistic regression, random forests (RF), artificial neural networks, and a novel deep-learning approach called Multi-graph Geometric Matrix Completion (MultiGMC). This research is significant because it explores how automated tools can support diagnostic decisions, particularly in “non-AVS” cases where traditional bedside examinations like Head Impulse, Nystagmus, Test of Skew (HINTS) may be less reliable.

A novel machine-learning framework was proposed that addresses the significant diagnostic challenges of dizziness and vertigo by integrating medical history with physical signs [17]. While previous automated tools typically relied on isolated data sources, this study utilized a retrospective dataset of 1003 patients across 16 distinct disease categories, including rare conditions such as Ramsay Hunt syndrome and hemodynamic orthostatic dizziness. The researchers extracted 31 attributes from medical history and 9 objective signs from bedside ocular motor examinations performed using video goggles. To process this multimodal data, they implemented Canonical Correlation Analysis (CCA) to establish a shared representation between history and signs, followed by an ensemble prediction model using soft-weighting and Gradient Boosting Decision Trees (GBDT).

A robust computational framework was introduced by Newman et al. for detecting and quantifying nystagmus within the massive datasets generated by the Continuous Ambulatory Vestibular Assessment (CAVA) device [18]. This wearable technology marks a paradigm shift in vestibular diagnostics by enabling continuous, 30-day ambulatory monitoring of eye movements via electronystagmography (ENG) to capture episodic dizziness events that are often missed during brief clinical visits. To analyze the resulting data, the researchers developed a multi-stage ensemble classification system incorporating Support Vector Machines (SVM), Linear Discriminant Analysis (LDA), and boosted trees that utilizes Fast Fourier Transform (FFT) to exploit the periodic spectral signature of nystagmus waveforms. The pipeline is further refined through a sieve filter to smooth outputs and a Dynamic Programming (DP) validation step that compares candidate signals against ideal sawtooth templates to minimize false positives caused by physical activities like running. In a blinded trial involving 17 healthy participants using VR-induced nystagmus, the proposed algorithm demonstrated exceptional diagnostic performance, achieving a recognition accuracy of 98.77%.

Nicholas Yang and colleagues address the clinical challenge of distinguishing canalithiasis Benign Positional Vertigo (BPV) from its mimics, such as vestibular migraine and posterior fossa tumors, which often present with similar positional nystagmus [19]. The study utilized a retrospective dataset of 476 video-nystagmography recordings to extract slow-phase velocity (SPV) profiles, focusing on six key temporal metrics: onset latency, 50% rise time, peak velocity, peak latency, and the time to 50% (T50) and 95% (T95) decay. The results highlight that machine learning (ML) models, particularly CatBoost, significantly outperformed traditional inferential statistical methods, achieving an accuracy of 93.3% compared to the 87.4% accuracy of the best performing statistical metric, T95. Explainability analysis using SHAP and Permutation Feature Importance confirmed that the models prioritized vertical peak SPV and T95 decay times, aligning with established clinical observations that BPV typically exhibits higher peak velocities and rapid decay.

In this study, the researchers evaluated the utility of five supervised machine learning (ML) algorithms—random forest, AdaBoost, gradient boosting, support vector machine (SVM), and logistic regression—to classify patients into peripheral vestibular (PV) and non-PV disease categories [20]. Using a dataset of 1009 patients who underwent 16 standardized neuro-otological examinations, the study extracted 44 clinical features, including results from caloric tests, stabilometry, and eye-tracking tests. The primary objective was to determine if these computational models could effectively support human decision making in the complex diagnostic process for vertigo and dizziness, which often involves a wide range of potential etiologies. The caloric test was identified as the most significant feature for classification across multiple models. Chaturvedi et al. provide a comprehensive systematic review of the application of machine learning (ML) and deep learning (DL) in diagnosing BPV through the analysis of videonystagmography (VNG) data [15]. The authors highlight that while BPV is a common and easily treatable cause of dizziness, it is frequently missed in frontline medical settings, such as emergency departments, due to a lack of specialist expertise in interpreting unique nystagmus patterns. By screening 3505 publications and retaining 23 key studies, the review evaluates how automated systems can act as expert surrogates, moving from traditional feature-based methods (like calculating slow-phase velocity) to sophisticated end-to-end deep learning architectures that process raw video data. The review identifies several critical barriers to clinical translation, most notably the scarcity of large, diverse, and publicly available datasets, which prevents robust benchmarking across different algorithms. Technical challenges such as sensitivity to ambient lighting, motion artifacts from abrupt head movements, and the “black box” nature of deep learning also hinder clinician trust and real-world adoption.

A systematic mini review of the role of artificial intelligence (AI) as a clinical auxiliary tool for diagnosing (BPPV) by Dai et al., has identified BPPV as the most prevalent peripheral vertigo disorder in clinical practice [21]. The review emphasizes that traditional diagnosis relying on the manual observation of nystagmus during positional provocative tests like the Dix-Hallpike and Roll tests is often limited by strong subjectivity and the significant physical demands placed on both patients and clinicians. To address these gaps, the authors examine the transition from manual interpretation to automated systems that utilize deep learning for complex feature extraction and pattern mining from nystagmus videos and objectively quantified ocular movement trajectories. The review highlights various high performing AI frameworks, such as ANyEye for robust pupil tracking and the “Look and Diagnose” (LAD) system, which aligns with clinical workflows by integrating binocular vision with body posture data. A significant trend identified in the review is the move toward multimodal fusion models, such as those combining eye movement video with normalized head position vectors, which have achieved diagnostic accuracies reaching 81.7%.

#### 2.1.2. Deep Learning Architectures for Nystagmus Detection, Quantification, and Pattern Recognition

ConVNG, a deep-learning framework designed to enable quantitative nystagmus assessment using standard smartphone video was validated to bridge the “care gap” caused by the high cost and resource intensity of gold-standard infrared video-oculography (VOG) goggles [12]. The authors fine-tuned a convolutional neural network (CNN) to track 17 facial and pupil landmarks, allowing for the extraction of granular kinematic data from 30 Hz RGB video. This framework was validated through a prospective study of healthy subjects using optokinetic stimuli and a retrospective analysis of clinical cases, demonstrating that it can operate without formal calibration protocols by relying on anthropomorphic assumptions. This makes ConVNG a highly accessible tool for frontline clinical triage or longitudinal at home monitoring where traditional equipment is unavailable. In benchmark comparisons, the framework demonstrated superior accuracy and precision over general purpose computer vision tools like Google’s MediaPipe. Furthermore, the model proved robust against real-world clinical variables such as facial masks and varying lighting, accurately analyzing retrospective clinical videos with relative deviations from VOG as low as 5%.

Vidith Phillips presents OpenNystagmus, an innovative open-source framework designed for the marker-free quantification of optokinetic nystagmus (OKN) using a standard smartphone [22]. Unlike previous smartphone-based applications that often required cumbersome head stabilization, fixed recording distances, or specialized goggles to maintain accuracy, this system is specifically engineered for hands-free and distance-agnostic deployment. The framework employs a sophisticated multi-stage processing pipeline that integrates traditional computer vision with deep learning; it utilizes Haar cascade classifiers for initial face detection, GroundingDINO for precise language-guided eye region localization, and EllSeg three-class segmentation to reconstruct the complete elliptical structures of the pupil and iris even during partial eyelid occlusion. The framework successfully captured physiologically consistent metrics, yielding horizontal gains (0.97–1.04) and vertical gains (0.72–0.74) that closely align with established normative values for the human oculomotor system while the researcher acknowledged a slight decrease in binocular detection yield during rapid ocular movements (from 89.2% to 79.6%).

Mun et al. propose a deep learning-based framework for the automated detection of nystagmus using video oculography (VOG) data to improve the diagnosis of BPPV [23]. Recognizing that traditional BPPV diagnosis is often subjective and time-consuming, the researchers developed a pipeline that first utilizes a 2D U-Net model for pupil segmentation and then extracts horizontal pupil movement as 1D time-series data. A significant technical contribution of this work is the implementation of a specialized “bridging algorithm” designed to handle missing data caused by blinks or poor tracking without inadvertently generating sawtooth patterns that could lead to false-positive nystagmus detections. This robust preprocessing ensures that the subsequent detection models operate on high-fidelity kinematic data derived from a large clinical dataset of over 3300 infrared videos. The authors conclude that for 1D time-series data of this nature, shallower models are more effective at extracting features while preventing the overfitting often seen in deeper, more parameter heavy networks.

A lightweight deep-learning framework was designed for real-time nystagmus tracking through the precise ocular object segmentation of the sclera, iris, and pupil [24]. The researchers trained their model using a diverse dataset of over 82,000 images, combining clinical video frames with synthetic eye images generated via virtual reality to maintain high accuracy even when clinical data is scarce. The architecture consists of two main modules: one dedicated to segmenting the eye regions and another for detecting blinks and calculating pupil center coordinates as 1D time-series data. Validation results demonstrate exceptional performance, with mean Intersection over Union (mIoU) values reaching 0.97 and a mean absolute tracking error of only 0.595, significantly outperforming benchmark models such as GazeML. A major strength of this framework is its superior execution speed, achieving up to 194 FPS on workstation-grade hardware and maintaining rapid performance on consumer-grade systems.

Yet another sophisticated deep learning framework, the Torsion-aware Bi-stream Identification Network (TBSIN), specifically engineered to detect the subtle and often imperceptible torsional eye movements associated with posterior semicircular canal pc-BPPV [25]. Recognizing that traditional videonystagmography (VNG) often lacks the granularity to capture these complex patterns, the authors developed a multi-stage pipeline that includes CNN-based video condensation to automatically remove invalid frames caused by blinking and a robust pupil calibration method that combines Circular Hough Transform with template matching to handle eyelash occlusion. A key technical innovation is the use of dense optical flow field analysis to create an optical flow guided torsion motion confidence (OFTMC) map, which allows the system to visually characterize the intensity and direction of torsional motion.

An automated framework was proposed for the classification of BPPV nystagmus patterns by integrating deep learning with dense optical flow [26]. Recognizing that traditional clinical examinations and existing video nystagmography (VNG) often struggle to objectively capture complex eye movements, particularly torsional nystagmus, the authors developed a five-stage pipeline to enhance diagnostic accuracy. The system includes an adaptive preprocessing method to remove invalid frames caused by blinking and a rapid iris-pupil segmentation module that avoids redundant eyelid data. At its core, the framework utilizes LiteFlowNet to extract motion features, which are then processed by a Nystagmus Video Classification Network (NVCN) that combines ConvNeXt for spatial feature extraction and LSTM for temporal integration. Experimental validation using a clinical dataset of 728 infrared videos demonstrates that the NVCN model is highly effective, achieving a 94.91% accuracy for general nystagmus pattern classification and 97.75% accuracy for specifically identifying torsional movements. A significant technical highlight is the speed of the proposed adaptive iris coarse segmentation method; it processes videos in just 1.17 s each, nearly 63 times faster than traditional transfer learning-based segmentation methods.

Lu et al. proposes a multimodal deep learning-based diagnostic model for BPPV that addresses the limitation of traditional single-modality AI tools by integrating eye movement video with 3D head position data [27]. The architecture utilizes a Big Kernel Temporal Difference Network (BKTDN) to extract sparse spatio-temporal features from high-resolution eye videos, employing large convolution kernels (up to 13 × 13) to enhance the model’s receptive field for detecting fine ocular movements. Simultaneously, a self-encoder abstracts normalized rotation angles of the major and minor axes into head position feature vectors, which are then fused with the oculomotor data using a cross-attention mechanism to simulate the complex reasoning process of a human physician.

Lee et al. introduce ANyEye, a nystagmus extraction system designed to automate the diagnosis of BPPV by analyzing video-nystagmography data [28]. The authors address a significant clinical bottleneck: while video-nystagmography is the non-invasive standard for observing characteristic eye movements, its interpretation currently relies on a limited number of trained specialists and is prone to errors from goggle slippage. To solve this, the researchers developed a convolutional neural network (CNN) framework that tracks the pupil trajectory in the challenging dark-field environments of clinical testing. A standout feature of the methodology is the integration of a slippage detection algorithm based on two-stage moving averages, which effectively filters out motion artifacts caused by rapid head movements or the heavy weight of the recording device, ensuring that only true nystagmus patterns are analyzed. The system outperformed both traditional iterative algorithms and other state-of-the-art CNN-based trackers, achieving a 91.26% detection rate at a five-pixel error threshold. This high level of precision allows for the detailed tracking of nystagmus waveforms including amplitude, speed, and direction which are critical for identifying specific BPPV subtypes.

A robust deep learning framework to automate the diagnosis of vestibular dysfunction through the analysis of horizontal nystagmus was presented [29]. The core methodology involves a three-tier system: a preprocessing module to remove obstructive frames like blinks, a motion trajectory module using the Segment Anything Model (SAM) for precise pupil localization, and a multi-scale 1-D CNN for final classification. Notably, the authors innovate by using a statistics-based prompt generation method to allow SAM to perform zero-shot pupil segmentation, effectively bypassing the need for expensive, manually annotated datasets typically required by traditional pupil trackers. This integration of high-level visual segmentation with temporal signal classification allows for a more focused analysis of pupil movement trajectories, which helps mitigate interference from background noise and environmental factors. The experimental results underscore the effectiveness of this approach, with the model achieving a precision of 81% for nystagmus identification, significantly surpassing existing 3D-CNN benchmarks.

A typical remote vestibular clinic requires a framework that addresses the limitations of existing BPPV diagnostic equipment through the integration of a wearable video capture device with advanced deep learning and signal processing techniques [30]. At its core, the system utilizes the Egeunet neural network, a lightweight U-shaped model chosen for its high segmentation accuracy and low memory consumption compared to standard Unet architectures. This model accurately segments eye structures such as the pupil and iris, which are then processed using a multi-fitting pupil localization (Mfp) algorithm to extract stable motion trajectories even in the presence of noise or deformations. Additionally, the framework incorporates Fast Fourier Transform (FFT) for intensity analysis and the Scale Invariant Feature Transform (SIFT) algorithm to detect iris feature points, enabling the measurement of horizontal, vertical, and rotational nystagmus components. In clinical evaluations, the framework demonstrated a high overall diagnostic accuracy of 93.33%, successfully identifying nystagmus triggering moments, directions, and amplitudes. A significant contribution of this research is the development of an intuitive diagnostic report that replaces traditional, difficult-to-interpret heuristic curves with clear visualizations, such as arrows representing individual nystagmus events.

A machine learning approach was explored to improve the diagnosis of BPPV, which is traditionally reliant on subjective and time-consuming manual clinical interpretation [31]. The researchers utilized a dataset of 2500 VNG recordings, from which they extracted frame segments and annotated them for the presence of nystagmus. The methodology involves using YOLOv11s for eye region segmentation to extract critical position, velocity, and shape features, which are then formatted into time-series data. This data is processed by a Long Short-Term Memory (LSTM) model, chosen for its proficiency in time-series classification, to categorize eye movements into three classes: normal (negative), horizontal nystagmus, and vertical nystagmus.

Lim et al. address the diagnostic challenges of BPPV, a common vestibular disorder that requires the precise interpretation of nystagmus, which can be difficult for non-specialists in primary care or emergency settings [32]. To assist in this process, the researchers developed a deep-learning diagnostic support system trained on an extensive dataset of 91,778 nystagmus videoclips from 3467 patients, all of which were annotated by four otology experts. The methodology involved extracting 30 spatiotemporal features representing three-dimensional eye movements across ten positional tests which were converted into grid images and fed into a 2D convolutional neural network (CNN) for classification.

A real-time framework for detecting Facial Movement Disorders (FMD) was introduced, specifically focusing on eyelid status (e.g., Orbicularis Myokymia and tics) and mouth droopiness, to assist in the diagnosis of neurological conditions such as stroke, multiple sclerosis, and Bell’s palsy [33]. The system leverages MediaPipe’s Face Mesh to track 468 facial landmarks and utilizes OpenCV for image preprocessing, operating through a user-friendly Streamlit-based web interface that supports standard laptop cameras, uploaded videos, or static images. A distinguishing technical feature is its “role-based” approach; instead of relying on resource-heavy deep learning training that requires significant computational power, the framework calculates Euclidean distances and relative ratios between specific ocular and oral landmarks and compares them against predefined experimental thresholds to identify irregularities. The researchers highlight that the system’s low computational requirements make it highly practical for real-time analysis in resource-constrained environments or primary care settings without the need for specialized hardware.

#### 2.1.3. Large Language and Vision Models as Zero-Shot Diagnostic and Triage Tools

Lacinski et al. presents a literature survey focused on leveraging artificial intelligence (AI) to enable Earth-independent medical care during long-duration space exploration missions, such as those to the Moon and Mars [34]. Recognizing that 40 min communication delays and limited resources make traditional ground support insufficient, the authors systematically evaluate AI tools applicable to 119 prioritized medical conditions identified by NASA’s IMPACT model. The review categorizes these technologies into ten systems-based domains ranging from general diagnostic chatbots and symptom mappers to specialized tools for respiratory, dermatologic, and vestibular health tracing the rapid evolution from foundational machine learning algorithms to sophisticated large language models (LLMs) like ChatDoctor and Almanac. While this survey addresses autonomous medical AI broadly rather than nystagmus diagnostics specifically, it is included here because vestibular health tracking is among the prioritized domains it evaluates, and it illustrates the zero-shot, resource-independent rationale that motivates LLM use in low-resource vestibular settings.

Noda et al. explore a novel approach to nystagmus classification by integrating a pupil-tracking algorithm with the GPT-4V large language model [35]. The researchers developed a system that extracts eye-movement trajectories from video recordings and converts them into either trace images or two-dimensional coordinate data (CSV files). Unlike traditional deep learning models that require vast training datasets, this method leverages GPT-4V’s in-context learning capabilities and varied prompting strategies, such as Chain of Thought (CoT) and Meta-Recognition (MR), to identify six types of nystagmus patterns.

The feasibility of using Gemini 2.0 for the automated detection of extraocular movement (EOM) disorders from real-world clinical videos [36] was evaluated. The researchers retrospectively analyzed 114 YouTube videos featuring conditions such as cranial nerve palsies, internuclear ophthalmoplegia (INO), supranuclear disorders, nystagmus, and ocular myasthenia gravis (MG), alongside 15 control videos of normal EOMs. The methodology involved using a standardized zero-shot prompt on videos that were trimmed to include only the pertinent clinical examination and stripped of audio cues. The model was assessed on its ability to provide a correct primary diagnosis, identify the affected eye (laterality), and recognize specific movement limitations. The study’s results indicate that while Gemini 2.0 shows promise in identifying overt abnormalities, its overall diagnostic accuracy was limited to 37.7%. Performance varied significantly by condition, with the model achieving high accuracy for third nerve palsy (81.1%), INO (80.0%), and sixth nerve palsy (66.7%), while correctly classifying 93.3% of normal cases. However, it struggled with more subtle presentations, such as ocular MG, where accuracy dropped to 20.0%.

### 2.2. Clinical Studies in Vestibular and Oculomotor Medicine

This original research investigates whether dimenhydrinate, an H1-receptor antagonist commonly used to suppress nausea and vertigo in acute unilateral vestibulopathy (UVP), hinders the brain’s natural compensation process [37]. The retrospective study compared patients who were prescribed the drug during hospitalization with those who were not, utilizing objective metrics from the video head-impulse test (VHIT) and rotatory chair test (RCT). Patients who took the suppressant showed less improvement in the vestibular ocular reflex gain for the posterior semicircular canal compared to the non-medicated group.

This case report describes a 33-year-old woman suffering from recurrent attacks of vertigo accompanied by fluctuating isolated high-frequency hearing loss [38]. This presents a diagnostic dilemma because the standard criteria for Meniere’s disease (MD) require documented loss in the low- to medium-frequency range. Despite the unusual frequency profile, her symptoms including tinnitus and ear fullness closely mimicked MD. Clinical examinations revealed a 44% unilateral weakness in caloric testing and persistent geotropic or ageotropic positional nystagmus during attacks.

This report details a rare case of a 79-year-old patient who developed persistent horizontal nystagmus following a hypoxic–ischemic brain injury [39]. Video-oculography showed exponentially increasing slow phases, a waveform type rarely observed in unconscious patients. Neuroimaging revealed delayed post-hypoxic leukoencephalopathy affecting the subcortical white matter and basal ganglia, while the brainstem and cerebellum appeared normal.

The landmark 1952 paper by Dix and Hallpike serves as a foundational text in neuro-otology by providing a rigorous clinicopathological framework to distinguish between three distinct types of organic vertigo: Meniere’s disease, vestibular neuronitis, and benign paroxysmal positional nystagmus [5]. Moving away from the ambiguous “Meniere’s syndrome” label, the authors utilized advanced functional tests and histological studies to define the unique pathological identities of these disorders. A key insight of the work is the definitive link between the loudness recruitment phenomenon and hair-cell disease in Meniere’s patients, supported by evidence of endolymphatic vesicle distension and structural degeneration in the organ of Corti. The authors identified vestibular neuronitis as a benign condition characterized by a lack of cochlear signs and a strong association with focal infections in the nose and throat. Furthermore, they redefined positional nystagmus by localizing the lesion to the otolith apparatus (the utricle and saccule) rather than the central nervous system. By detailing a specific maneuver to elicit paroxysmal, rotatory nystagmus involving briskly moving the patient into a critical supine position Dix and Hallpike established a diagnostic protocol that remains a clinical standard. Their findings were substantiated by histological evidence of chronic tissue changes in the utricular macula, providing a scientific basis for identifying which ear is affected during clinical examination.

The 2017 Clinical Practice Guideline: BPPV (Update), published by the American Academy of Otolaryngology—Head and Neck Surgery Foundation, provides a comprehensive, evidence-based framework for standardizing the care of BPPV, the most common vestibular disorder [7]. The update incorporates new evidence from 27 randomized controlled trials and 20 systematic reviews to refine diagnostic and therapeutic protocols. A central pillar of the guideline is the strong recommendation for the Dix-Hallpike maneuver as the gold standard for diagnosing posterior semicircular canal BPPV, emphasizing the identification of characteristic torsional, upbeating nystagmus. Furthermore, the guideline introduces the supine roll test as a critical secondary diagnostic step to identify lateral (horizontal) canal BPPV when Dix-Hallpike results are inconclusive, ensuring that less common variants are not overlooked. The guideline’s therapeutic recommendations prioritize efficiency and the reduction of unnecessary healthcare costs, making a strong recommendation for canalith repositioning procedures (CRP), such as the Epley maneuver, as initial therapy. In a significant shift from traditional practices, the update issues a strong recommendation against postprocedural postural restrictions, such as sleeping upright or wearing cervical collars, noting that they do not improve treatment efficacy and can cause musculoskeletal discomfort. Additionally, the guideline seeks to eliminate diagnostic waste by recommending against routine radiographic imaging, comprehensive vestibular testing, and the use of vestibular suppressant medications (like antihistamines or benzodiazepines) for patients who meet the clear clinical criteria for BPPV. By emphasizing shared decision-making and patient education regarding fall risks and recurrence, the paper aims to improve overall quality of life and patient safety.

This paper details a case of idiopathic opsoclonus in a 39-year-old woman who presented with acute vertigo, vomiting, and unsteady gait [40]. The clinicians observed high-frequency (15 Hz) and multi-directional “dancing” eye movements, which were recorded in real time using a smartphone during the acute attack. Although initial imaging and tests were normal, elevated IgG and IgM titers suggested a herpes simplex virus-related infection. The patient underwent a one-month treatment regimen involving intravenous and oral corticosteroids combined with clonazepam, which successfully eliminated the pathologic eye movements.

The study by Niels West and colleagues investigates the efficacy of mechanical repositioning chairs specifically the TRV chair in addressing the subjective and emotional burden of refractory BPPV [41]. Recognizing that BPPV can be a deeply debilitating condition often linked to psychological distress, the researchers conducted a prospective cohort study of 31 patients who had previously failed standard manual repositioning maneuvers. To quantify the “disease burden,” the study utilized three standardized self-assessment tools: the Dizziness Handicap Inventory (DHI) for functional impact, the Visual Analog Scale (VAS) for subjective vertigo intensity, and the Hospital Anxiety and Depression Scale (HADS) to track emotional well-being. The findings demonstrate that mechanical intervention significantly improves patient outcomes, with complete remission typically achieved in a mean of just two treatment sessions.

The Guidelines for Reasonable and Appropriate Care in the Emergency Department 3 (GRACE-3) provides a comprehensive, evidence-based framework for managing adult patients presenting to the emergency department (ED) with acute dizziness and vertigo [8]. Developed by a multidisciplinary panel using the GRADE approach, the paper addresses the significant diagnostic challenges and high costs associated with these visits, which often result in an over-reliance on inaccurate neuroimaging. A central theme of the guidelines is the rejection of the traditional “symptom quality” paradigm which focuses on how a patient describes their dizziness in favor of a more reliable “timing and triggers” approach. This framework categorizes patients into three clinical syndromes: Acute Vestibular Syndrome (AVS), Spontaneous Episodic Vestibular Syndrome (s-EVS), and Triggered Episodic Vestibular Syndrome (t-EVS), each dictating a specific diagnostic path. The guidelines offer 15 specific recommendations that prioritize bedside physical examination techniques over routine neuroimaging, noting that non-contrast CT has very low sensitivity for identifying acute ischemic strokes in dizzy patients. For patients with AVS, the panel recommends the HINTS (Head Impulse, Nystagmus, Test of Skew) examination for trained clinicians, while suggesting finger rub hearing tests and gait assessments as additional diagnostic aids. In cases of t-EVS, the Dix-Hallpike test is emphasized for diagnosing posterior canal BPPV, which should be treated immediately with the Epley maneuver.

BPPV is a mechanical disorder of the peripheral vestibular system caused by displaced otoconia, most frequently affecting the posterior canal [42]. The paper by Power et al. emphasizes that diagnosis relies on characteristic nystagmus elicited through the Dix-Hallpike maneuver (DHP) or Supine roll test (SRT). A significant clinical finding is the necessity of repeated testing within a single clinical session if a patient’s history strongly suggests BPPV, as approximately 13% of patients in the study required up to three DHP tests before a positive result was obtained. This approach is promoted as more efficient than multi-session testing, as it helps avoid false negatives and reduces the duration of symptoms and associated fall risks. Treatment via Canalith Repositioning Procedures (CRP) was found to be highly effective, with 91% of posterior canal and 88% of horizontal canal cases resolving in two or fewer maneuvers.

A retrospective study investigated the incidence of Lateral Semicircular Canal LSC BPPV among 273 patients who were initially diagnosed with vestibular neuritis (VN) in an Emergency Department (ED) setting [43]. The preliminary VN diagnoses were based on the presence of persistent horizontal nystagmus in an upright position, intense vertigo, and negative neurological evaluations. By performing the Head Pitch Test (HPT) while patients were seated, researchers discovered that 56 of these patients (approximately 20.5%) actually had LSC BPPV. This group included 37 geotropic and 19 apogeotropic variants, all of whom were successfully treated during the same session using liberatory maneuvers such as the Vannucchi-Asprella or Gufoni maneuvers. The paper emphasizes that Pseudo-Spontaneous Nystagmus (PSN) is the primary reason LSC BPPV “masquerades” as VN; it appears as a long-lasting horizontal nystagmus in the upright position that mimics the spontaneous nystagmus of a vestibular loss. However, unlike the direction-fixed nystagmus of VN, PSN is gravity-sensitive and changes its beating direction or intensity when the head is tilted forward or backward in the pitch plane.

A different study details the significant challenges space exploration poses to human balance and orientation, primarily due to microgravity [34]. In space, the otolith organs are “unloaded” and fail to provide the brain with a world-based gravitational reference, leading to spatial disorientation, illusory sensations of inversion, and impaired gaze stabilization. While astronauts demonstrate robust sensorimotor adaptation during short missions, recovery upon returning to Earth can take weeks or months following long-duration travel. The review also emphasizes the overlooked danger of space radiation, such as galactic cosmic rays and solar particle events. Contrary to the traditional belief that the temporal bone is resistant to radiation, evidence from animal models and human radiotherapy indicates that both the vestibular apparatus and central processing pathways are vulnerable to damage.

This article provides a comprehensive overview of hemodynamic orthostatic dizziness (OD), which results from global cerebral hypoperfusion triggered by standing [17]. It outlines the diagnostic criteria for conditions like orthostatic hypotension (OH) and postural tachycardia syndrome (POTS), emphasizing the use of autonomic function tests, such as the head-up tilt test and Valsalva maneuver. The paper notes that OD is common across all ages and is a significant contributor to non-vestibular dizziness diagnoses. Management of hemodynamic OD is highly individualized, focusing on alleviating symptoms and preventing falls rather than just reaching specific blood pressure targets.

This prospective randomized controlled trial evaluates treatment modalities for acute low-tone sensorineural hearing loss (ALHL), a condition often associated with cochlear hydrops [44]. The study compared the efficacy of a combined regimen of methylprednisolone and the osmotic diuretic isosorbide against a steroid-only control group. Hearing outcomes were measured by absolute gain at low frequencies over an eight-week follow-up period. While both groups showed high recovery rates of approximately 86.7%, the combined therapy group demonstrated higher absolute hearing gains at 125, 250, and 500 Hz.

This observational study investigates the mechanism behind dizziness caused by cholinesterase inhibitors (ChEIs) like donepezil, which are widely used to treat Alzheimer’s disease [45]. Using video-oculography, researchers recorded the eye movements of three patients who developed unsteadiness following the initiation or dose increment of their medication. The study found that all three patients exhibited frequent saccadic oscillations (specifically square wave jerks) that disrupted visual fixation and induced dizziness. These abnormal movements markedly decreased or resolved entirely after the medication was discontinued.

Melliti et al. investigate the interobserver and intraobserver agreement among six vestibular medicine specialists diagnosing BPPV using only eye movement recordings from 240 patient cases [46]. The study found that overall interobserver agreement was only fair to moderate, with a Cohen’s kappa value of 0.40 and a 60% proportion of agreement. While intraobserver agreement reached “almost perfect” levels for one individual, the general consensus across different experts remained relatively low. These results highlight the inherent challenges and variability in diagnosing complex vestibular disorders when clinicians are limited to visual data without broader clinical context. The authors attribute this suboptimal agreement primarily to the absence of supplementary clinical information such as patient history, head position data, and real-time feedback during positional maneuvers [46].

Sugita et al. investigated the functional differences between retinal ON and OFF pathways in driving the initial optokinetic responses (OKRs) in mice [47]. By utilizing two-frame motion stimuli separated by varying interstimulus intervals (ISIs), the researchers aimed to estimate the temporal filters of the visual system. Wild-type mice were compared with TRPM1 (transient receptor potential cation channel, subfamily M, number 1) mutants, the latter of which possess dysfunctional ON bipolar cells, effectively isolating the OFF-pathway’s contribution to behavior. The methodology relied on the phenomenon where increasing the ISI leads to a directional reversal of eye movements, a result of the biphasic nature of the temporal filters involved in motion detection. The findings reveal that while both mouse types exhibited directional reversals as ISIs increased, TRPM1^−/−^ mice showed reversal at significantly shorter ISIs (26.7–53.3 ms) compared to wild-type mice (∼106.7 ms). Model simulations indicated that temporal filters in TRPM1^−/−^ mice are tuned for higher frequencies and exhibit faster dynamics, characterized by a significantly shorter time-to-peak in their response. This suggests that the OFF pathway provides high-temporal resolution signals via ionotropic receptors, whereas the ON pathway contributes slower dynamics through metabotropic receptors, enhancing responses to low-frequency visual motion.

### 2.3. Smartphone and Portable Hardware Innovations

A study details the development of a smartphone-based application designed to detect and qualify nystagmus, a critical symptom for differentiating between acute central emergencies, such as strokes, and peripheral vestibular disorders [11]. Developed for the Android platform using the OpenCV framework and an eye-tracking algorithm, the app aims to provide a rapid, cost-effective diagnostic tool for emergency medical settings and telemedicine. The researchers validated the software through a prospective study of twenty healthy participants, using caloric irrigation and optokinetic stimulation to provoke nystagmus. This experimental design allowed the team to compare the app’s performance in identifying the position (horizontal vs. vertical) and direction of eye movements against the “gold standard” of video-computer nystagmography.

One of the review articles on portable hardware innovations explores the diagnostic necessity of eye movement assessment for identifying vestibular disorders and differentiating between peripheral issues and central emergencies like strokes [9]. While infrared video-oculography (VOG) goggles are the current gold standard, their widespread use is hindered by high costs reaching up to USD 40,000 and the requirement for trained technicians to operate complex software. To address these limitations, the authors highlight smartphone-based applications as a cost-effective and ubiquitous alternative. By utilizing augmented reality features and advanced cameras, smartphones can track pupil position and head movement with high correlation to traditional goggles. Parker et al. focus on evaluating the accuracy and precision of eye and head tracking using a custom smartphone application built on Apple’s ARKit framework [10]. The study’s core objective was to determine if this “EyePhone” app, which utilizes infrared and natural light sensors to track position data at 60 samples per second, could provide a more accessible quantitative alternative to expensive, “gold-standard” video-oculography (VOG) goggles. To validate the application, the researchers enrolled 12 healthy volunteers and performed three specific experiments involving eye-only, head-only, and combined movements toward targets at known eccentricities on a wall.

Hartness et al. provided a comprehensive systematic review and meta-analysis of automated strabismus evaluation technologies, spanning literature published from 1949 to 2025 [48]. The study screened 69 articles and conducted a statistical meta-analysis on 17 of them to determine the correlation between automated measurements and gold-standard clinical assessments, such as the alternate prism cover test (APCT). The researchers found a strong pooled correlation of 0.87 between the two methods, indicating that diverse technologies ranging from static photographic analysis and automated Hirschberg tests to sophisticated video-based pupil tracking and virtual reality headsets show significant clinical promise. This analysis underscores the transition toward objective, image-based quantification to reduce subjective variability and expedite the diagnostic workflow in busy clinical environments. Despite the strong statistical agreement, the authors identify several critical barriers that hinder the widespread clinical adoption of these automated tools. Although automated strabismus evaluation is an adjacent oculomotor application rather than a nystagmus or vertigo task, it is included because its image-based quantification methods, reference-standard comparisons, and clinical-adoption barriers closely parallel those facing automated nystagmus assessment.

A pilot study validating a lightweight, visible-light eye-tracking algorithm was designed for smartphone deployment to facilitate the early screening of neurodegenerative diseases [49]. The research compares two specific frameworks: CHT TM, which integrates Circular Hough Transform with Template Matching, and CHT ACM, which utilizes Active Contour Models. By using an iPhone 12 to record subjects with varying iris colors performing vertical, horizontal, fixation, and circular tasks, the authors developed a computationally efficient alternative to resource-heavy deep learning models. This approach prioritizes edge computing, allowing for real-time analysis directly on the device to maintain user privacy and reduce latency in low-resource medical settings. This work targets neurodegenerative screening rather than vestibular diagnosis directly but is relevant here as one of the few visible-light, edge-computing eye-tracking pipelines validated on a smartphone, demonstrating a hardware and processing approach transferable to nystagmus quantification.

High-accuracy screening method for intermittent strabismus using a low-cost, wearable eye-tracking device combined with AI-enhanced ocular analysis [50] was evaluated. Traditional clinical methods, such as the alternating prism coverage test (PCT), often suffer from subjective error and struggle to capture the erratic, occasional eye misalignments characteristic of early-stage or intermittent strabismus. To address this, the researchers developed a GBP 40 3D-printed device equipped with near-infrared cameras that capture spatially continuous high-definition images during a wide-angle visual induction paradigm. This approach is particularly significant because it does not require a manual eye-occlusion process, making it highly suitable for large-scale screening in non-clinical environments like schools or community centers where medical resources may be limited. In a study involving 70 subjects, the system achieved a 97.1% accuracy rate, demonstrating its potential to facilitate timely intervention and prevent permanent vision loss.

Phillips et al. present a successful proof-of-concept for EyePhone, an in-house smartphone application designed to detect positional nystagmus, the clinical hallmark of BPPV [51]. The research addresses a critical gap in vestibular care: while BPPV is a leading cause of dizziness, it is frequently misdiagnosed in emergency settings, leading to the overuse of expensive, low-yield imaging. To solve this, the authors utilized Apple’s ARKit API to capture high-definition eye-tracking data (1080p at 60 fps) during standard Dix-Hallpike and supine roll maneuvers. By employing an embedded MATLAB R2022a algorithm to extract the velocity of slow-phase eye movements and apply a hard threshold for detection, the system provides an objective, quantified alternative to traditional visual observation. In a trial involving ten participants and 23 positional test recordings, the EyePhone app achieved a 100% accuracy rate when compared against an adjudicated gold standard of expert clinician review. The system demonstrated 100% sensitivity and specificity, correctly identifying all instances of nystagmus while ruling out the condition in all negative traces.

Bastani et al. demonstrate the high accuracy of EyePhone, a smartphone-based application, in detecting and quantifying induced optokinetic nystagmus (OKN) compared to the clinical standard of video oculography (VOG) [52]. By utilizing an iPhone’s front-facing camera and the ARKit API, the system captured high-definition eye position data from healthy volunteers as they viewed visual stimuli moving at incremental velocities in four directions. The researchers employed a MATLAB-based algorithm to extract slow-phase velocities (SPV), finding that EyePhone’s measurements highly correlated with VOG recordings (*r* = 0.98 for horizontal and *r* = 0.94 for vertical nystagmus). This correlation proves that ubiquitous smartphone hardware can provide precise oculomotor analysis similar to expensive, specialized VOG goggles. Sefein et al. present the development and clinical evaluation of the Portable System for Capturing Nystagmus (PSCN), designed as a cost-effective and mobile alternative to the expensive, office-based Video Nystagmography (VNG) [53]. The system consists of a modified goggle equipped with a Raspberry Pi camera and a microcontroller that captures eye movements and analyzes them using an optical flow algorithm. This algorithm tracks the pupil by detecting the vertical edge of the limbus, a method preferred for its accuracy even with darker irises. The processed data is transmitted via Bluetooth to an Android application, which displays results in a user-friendly graph form, allowing for quick interpretation and remote diagnostic capabilities. In clinical and laboratory testing, the PSCN demonstrated high reliability and an accuracy ratio of up to 80% when compared to the gold standard VNG. Notably, the system’s software successfully differentiated real nystagmus from artifacts like eye blinking by removing noise during data processing. Patient feedback indicated that the wearable device was tolerable in both weight and size.

Another novel proof-of-concept called “VertiScope,” an oculography system that utilizes a smartphone-based eye-tracking algorithm to assess optokinetic nystagmus (OKN) [54]. To ensure high-quality data collection, the researchers developed a custom head stabilization device known as the “Precision View Stand”, which incorporates a white LED circuit for consistent illumination and a chin rest to minimize motion artifacts during recordings. The system’s software employs a centroid-based tracking algorithm to identify the pupil-iris complex and calculate the slow-phase velocity (SPV) of horizontal nystagmus. This portable technology is designed to overcome the limitations of traditional, office-bound VNG by enabling ictal monitoring testing during active vertigo symptoms and increasing accessibility in resource-limited or non-specialized clinical settings. In a prospective study involving 39 healthy participants, VertiScope demonstrated high reliability and accuracy when compared to the gold-standard VNG system. The mean SPV measured by the smartphone device (22.13 ± 5.26°/s) was not significantly different from that recorded by the reference VNG (23.66 ± 5.21°/s), showing a strong correlation between the two methods.

Özel introduces a technical innovation aimed at making vestibular evaluation more accessible: a 3D-printed Smartphone Video-Frenzel Device (SVFD) [55]. By utilizing the macro camera and integrated flashlight of a modern smartphone, the device effectively suppresses ocular fixation (OF) a critical requirement for observing peripheral nystagmus while providing high resolution color video recordings. The design is notably practical, featuring a magnetic mounting system (compatible with both MagSafe and non-MagSafe phones) and a dual-strap head fixation that ensures stability during positional testing. This approach offers a low-cost, portable alternative to expensive, non-portable videonystagmography (VNG) systems, which are often limited to specialized centers.

A portable alternative to conventional video-oculography (VOG) is introduced through wearable augmented reality (AR) for nystagmus examination [56]. The system utilizes J7EF Gaze smart glasses equipped with infrared eye-tracking sensors and dual Si-OLED displays to deliver standardized oculomotor stimuli, such as saccades and smooth pursuit, via an Android-based portable device. In a randomized crossover design involving patients with vertigo, the AR system was compared directly to VNG Ulmer protocols to evaluate its usability, accuracy, and tolerability in a clinical hospital environment. The results indicate that the AR-based system achieved a diagnostic accuracy of 77.1%, with a particularly high negative predictive value (93.3%), making it effective for ruling out central vestibular abnormalities. Patients reported no significant discomfort, confirming that the wearable device is as tolerable as traditional VOG.

### 2.4. Telemedicine and Remote Monitoring

Sanghvi et al. introduce a proof-of-concept AI-driven telehealth framework designed for the marker-free detection and quantification of nystagmus, particularly to support clinical decision-making in remote settings [14]. The system utilizes a cloud-based technology stack featuring Python, OpenCV, and MediaPipe’s Face Mesh to track 468 distinct facial landmarks in real time from standard smartphone videos. The automated workflow allows a caregiver to record a video focusing on the patient’s eyes, which is then uploaded to the cloud for the AI to calculate slow-phase velocity (SPV) and generate visual graphs for professional review. This physician-focused model ensures that while data collection is automated to reduce subjective variability, the final diagnostic interpretation and management recommendations remain with a clinician. The framework’s validation against “gold-standard” videonystagmography (VNG) demonstrated exceptional performance, achieving an accuracy of 98% with a remarkably low mean squared error of 0.00459. Statistical analysis confirmed that the AI-calculated SPV measurements were equivalent to VNG outputs, showing a correction error margin of only ±4.8%.

aEYE, a deep-learning system is designed to democratize the detection of nystagmus by enabling non-experts to identify this critical symptom through automated video analysis [57]. Unlike traditional eye-tracking methods that require high-fidelity measurements of the pupil center or iris features, aEYE utilizes a filtered image-based motion classification approach. This technique transforms grayscale video frames into a representation of ocular motion, allowing the system to classify segments as “nystagmus” or “no nystagmus” without needing precise pixel-to-angle calibration. The framework was developed and validated using a subset of monocular video-oculography (VOG) recordings from the AVERT clinical trial, providing a dataset derived from real-world patients presenting with acute dizziness and vertigo. A key finding of the study is that the system remains effective even under degraded video conditions; while accuracy decreases as sampling rates drop from 60 Hz to 15 Hz, it is remarkably resilient to lower image resolutions, making it highly suitable for telemedicine applications where bandwidth is limited.

iCapNYS, an innovative smartphone-based system is designed to record eye movements and head positions during spontaneous vertigo attacks, regardless of location or time [58]. This system directly addresses a major clinical hurdle: the transient nature of vertigo, which often results in the absence of observable symptoms by the time a patient reaches a medical facility for an appointment. A unique “eco-friendly” feature of the methodology is the implementation of recyclable cardboard goggles that securely hold an iPhone and block out surrounding visual stimuli. This design is critical as it prevents gaze-induced nystagmus suppression, allowing for the stable recording of spontaneous eye movements in a non-fixating state. To ensure accessibility for older adults, the app’s interface is simplified to require only three taps to launch and complete a recording. Technical validation of the iCapNYS system was demonstrated through a comparative analysis with the clinical standard of infrared video Frenzel glasses in an 82-year-old patient experiencing an active Meniere’s disease attack. The results indicated that the recorded nystagmus characteristics including frequency and slow-phase velocity were highly comparable, with the app recording a velocity of 12.0°/s compared to the clinical standard’s 12.6°/s. By allowing patients to email these recordings directly to healthcare providers, the system supports telemedicine and may facilitate timely interventions, such as self-treatment maneuvers for BPPV.

Sakazaki et al. present a cost-effective, patient-initiated monitoring system designed to capture nystagmus during active vertigo attacks at home [59]. This innovation addresses a significant diagnostic gap where the transient nature of episodic vertigo often leads to the resolution of symptoms before a patient can be evaluated in a clinical setting. The researchers developed a system consisting of a commercially available mini-infrared camera costing approximately USD 25 and 3D-printed goggles produced for about USD 13 in filament costs. By using a paired smartphone app to control the camera via Wi-Fi, patients can record high-definition eye movements in complete darkness, which is critical for preventing the suppression of nystagmus by visual fixation. This low-cost framework offers a scalable solution for telemedicine, potentially saving healthcare systems significant costs by reducing unnecessary tests and streamlining the diagnostic process. The utility of the device was validated through a case report of a 40-year-old male whose initial clinical evaluations using standard Frenzel glasses revealed no abnormalities. Using the 3D-printed system at home, the patient successfully recorded geotropic direction-changing positional nystagmus during a vertigo episode, leading to a definitive diagnosis of lateral semicircular canal-type BPPV. This diagnosis was subsequently confirmed when the patient’s symptoms were effectively alleviated using the Gufoni maneuver.

Patil et al. address the suboptimal diagnostic accuracy and high healthcare expenditures associated with vertigo by exploring novel risk stratification metrics, clinical pathways, and technological tools [60]. The paper emphasizes the critical need for clinicians to distinguish between peripheral etiologies, such as BPPV, and potentially life-threatening central etiologies like stroke. By evaluating traditional bedside maneuvers alongside modern interventions, the authors highlight that while neuroimaging is often overused, low-cost physical examinations like HINTS and the Dix-Hallpike maneuver remain underutilized in primary and emergency care settings. The review suggests that systematic implementation of standardized algorithms and targeted provider education can significantly reduce unnecessary diagnostic tests and hospital stays without compromising patient safety.

## 3. Analysis

### 3.1. AI-Driven Nystagmus Analysis

AI-driven nystagmus and vertigo diagnosis have been approached through two principal paradigms classical machine learning, which relies on engineered features such as slow-phase velocity, and deep learning, which operates directly on pupil trajectories extracted from video-oculography recordings. As summarized in Table 1, reported performance spans accuracies from roughly 76% for single classifiers to over 98% for ensemble and multimodal approaches, though direct cross-study comparison remains limited by heterogeneous datasets, differing cohort sizes, and inconsistent validation protocols.

#### 3.1.1. Machine Learning Approaches

Classical machine learning applied to vestibular diagnostics addresses two primary task categories: binary classification of central versus peripheral etiologies, and multi-class differentiation across broader disease spectra. Dataset scales range from 108 emergency department patients [13] to 1009 patients evaluated across 44 clinical features [20], with the broadest disease coverage provided across 16 diagnostic categories in 1003 patients [17]. Five supervised classifiers—random forest, AdaBoost, gradient boosting, SVM, and logistic regression are benchmarked on 1009 patients, finding individual accuracies of 76–79% with SVM performing best in isolation [20]. A consistent cross-study finding is that ensemble and combination strategies outperform single-algorithm approaches: accuracy rises to 83–85% when all five models agree [20]; RF integrated with AdaBoost achieves 97% accuracy and AUC of 0.95 on a 294-patient vertigo triage dataset [16]; and combining SVM, LDA, and boosted trees with Fast Fourier Transform-based spectral features achieves 98.77% nystagmus recognition accuracy in 30 day ambulatory recordings the highest reported in the ML subset [18].

Feature engineering and clinical interpretability are central analytical concerns. Slow-phase velocity (SPV) is independently identified as the most discriminative feature by both Lai et al. and Yang et al. through SHAP analysis [16,19], reinforcing its established physiological significance. The SPV temporal profile is further decomposed into six metrics onset latency, 50% rise time, peak velocity, peak latency, T50, and T95 decay across 476 VNG recordings, with CatBoost achieving 93.3% accuracy in separating canalithiasis BPV from mimics, outperforming the best single statistical threshold by approximately 6 percentage points [19]. Feature modality integration is demonstrated as equally critical in a retrospective dataset of 1003 patients, where combining medical history with physical signs via Canonical Correlation Analysis and Gradient Boosting Decision Trees yields 98.11% accuracy and F1 of 95.43% substantially higher than either modality alone with stability maintained when physical sign data is perturbed to simulate inexperienced clinician input [17].

The clinically most consequential binary task differentiating vestibular stroke from peripheral neuritis is most rigorously benchmarked against HINTS and ABCD2 clinical scores across multiple ML architectures on 108 ED patients [13]. The multi-graph geometric matrix completion model achieves AUC 0.96 versus 0.71 for HINTS, with the performance gap widening further for non-AVS presentations where HINTS drops to near chance AUC of 0.54 while ML models remain robust. The field-wide constraints that persist across ML approaches including single institution datasets, absence of external validation, class imbalance for rare BPPV subtypes, and lack of standardized feature sets are confirmed across systematic reviews of this domain [15,21], limiting cross-study comparability and restricting clinical translation.

#### 3.1.2. Deep Learning Approaches

Reliable pupil localization under clinical conditions is the foundational technical challenge across all deep learning frameworks, with occlusion, blink artifacts, varying illumination, and goggle slippage addressed through divergent architectural strategies. A U-Net with ellipse-fitting compensation and a two-stage moving average slippage detection algorithm achieves 91.26% detection at a five-pixel error threshold [28]. Tri-class segmentation of sclera, iris, and pupil, trained on over 82,000 images combining clinical and synthetic VR-generated data, achieves mIoU of 0.97 and up to 194 FPS on workstation hardware [24]. The Segment Anything Model with statistics-based prompt generation enables zero-shot pupil segmentation at 79.53% detection within a 10-pixel error margin on the LPW benchmark, bypassing the requirement for manually annotated training data entirely [29]. The lightweight Egeunet combined with multi-fitting pupil localization, FFT-based intensity analysis, and SIFT iris feature tracking simultaneously captures horizontal, vertical, and rotational nystagmus components [30]. The lowest reported calibration error in the entire AI corpus 0.38°, significantly below the 1° clinical threshold is achieved through a three-stage pipeline integrating Haar cascade classifiers, GroundingDINO for language-guided eye region localization, and EllSeg three-class segmentation on standard RGB smartphone video without formal calibration protocols [22].

The dominant pipeline architecture extracts 1D time-series pupil trajectories from segmented frames and applies temporal classifiers for nystagmus detection and classification. On over 3300 infrared VOG videos, a custom CNN1D outperforms ResNet and GoogLeNet at 91.02% accuracy and F1 of 92.68%, with the critical contribution being a bridging algorithm that reconstructs missing tracking data during blinks without generating false sawtooth artifacts that would trigger false-positive detections [23]. The largest annotated training corpus in the deep learning subset—91,778 video clips from 3467 patients annotated by four otology experts yields sensitivity and specificity exceeding 87% for horizontal and vertical nystagmus detection, with torsional accuracy (sensitivity 0.783) remaining lower due to iris resolution constraints at standard capture resolutions [32]. Combining YOLOv11s eye region segmentation with LSTM classification on 2500 VNG recordings yields 82% overall accuracy, with vertical nystagmus classified most reliably due to its low baseline occurrence making pathological patterns more discriminable from normal horizontal gaze behavior [31].

Torsional nystagmus detection presents the greatest technical challenge and has motivated dedicated architectural development due to its clinical necessity in posterior canal BPPV diagnosis. The Torsion-aware Bi-stream Identification Network employs dense optical flow to generate torsion motion confidence maps, achieving 85.73% frame-level accuracy and 67.45% IoU for segment localization [25]. Extending optical flow with LiteFlowNet-extracted features through a ConvNeXt-LSTM Nystagmus Video Classification Network achieves 94.91% general and 97.75% torsional classification accuracy, the highest for this subtype in the corpus while reducing per-video processing time to 1.17 s, approximately 63 times faster than transfer learning-based segmentation baselines [26]. A CNN fine-tuned on 17 facial and pupil landmarks extracted from 30 Hz RGB smartphone video produces SPV measurements statistically equivalent to gold-standard VOG goggles with deviation as low as 5%, demonstrating that clinical equivalence does not require infrared hardware [12]. Extending the facial landmark paradigm to 468 MediaPipe Face Mesh landmarks for eyelid and mouth droop detection, a threshold-based distance ratio approach achieves 93–95% accuracy without deep learning training [33].

Multimodal fusion of eye movement and head position data represents the most architecturally significant advance in the deep learning subset. The contribution of head position data is quantified directly through a Big Kernel Temporal Difference Network fused with head position feature vectors via cross-attention, achieving 81.7% accuracy on 518 BPPV patients but collapsing to 39.4% when head position is removed [27] a 42-percentage-point drop establishing postural context as a necessary rather than supplementary input for BPPV classification. This finding has structural implications for all single-modality nystagmus-only frameworks, whose performance ceilings may reflect the inherent ambiguity of eye movement data absent postural context. Across the full deep learning corpus, the common unresolved limitations are dataset scarcity for anterior canal BPPV and cupulolithiasis subtypes, sensitivity to ambient lighting and motion artifacts in uncontrolled environments, and the absence of explainable AI mechanisms that would support clinician trust and regulatory approval [15,21].

#### 3.1.3. Large Language Models and Vision Models

The deployment of large language and vision models for nystagmus and ocular motility analysis represents the earliest-stage development within the AI subsections reviewed, and current performance is substantially below that of task-specific deep learning. GPT-4V evaluated on six nystagmus pattern types using Chain-of-Thought and Meta-Recognition prompting strategies yields overall classification accuracy of 17–38% well below the 80%+ benchmark of CNN and LSTM models [35]. Horizontal nystagmus achieves the best per-type accuracy at 69%, while vertical and torsional components are poorly resolved due to smaller movement amplitudes and the inherent limitation of 2D trajectory representation. Gemini 2.0 assessed on 114 clinical videos of extraocular movement disorders under zero-shot prompting reports 37.7% overall accuracy [36], with condition-specific performance ranging from third nerve palsy (81.1%) and internuclear ophthalmoplegia (80.0%) both visually overt and anatomically localizable down to ocular myasthenia gravis at 20.0%, reflecting the difficulty of subtler functional disorders. In both studies, correct diagnosis correlates strongly with accurate identification of specific movement limitations and laterality, suggesting that explicit spatial reasoning remains a current bottleneck for vision models in oculomotor analysis. These tools are most appropriately framed as experimental aids for triage, documentation, and decision support rather than as standalone diagnostic systems.

Despite low classification accuracy, LLMs and vision models offer a structurally distinct operational advantage: zero-shot inference requiring no task-specific training data. This is directly relevant to rare vestibular conditions and low-resource environments where annotated datasets are unavailable or prohibitively expensive. A survey of LLM-based tools across 119 NASA-prioritized conditions contextualizes this advantage within the broader demand for autonomous AI-driven medicine in space exploration [34], identifying a consistent pattern: LLMs can approach physician-level accuracy on structured clinical vignette questions but fall short of autonomous management of acute vestibular disorders requiring real-time sensorimotor data. The consensus across all three papers is that LLMs and vision models are best positioned as first-pass triage tools, documentation aids, and educational resources within a supervised clinical decision support architecture augmenting specialist AI or human expertise rather than replacing it.

### 3.2. Clinical Evidence Analysis: Progress, Tensions, and Diagnostic Ambiguities

**Figure 2:**
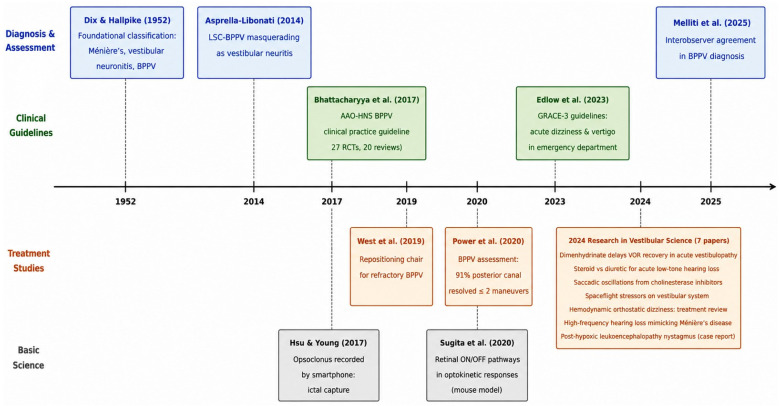
Chronological timeline of popular vestibular diagnostic approaches reviewed, divided into diagnostic milestones and treatment milestones. Each node indicates the paper, year, and primary contribution to vestibular clinical practice.

The medicine-based papers span seven decades, from the 1952 foundational pathology work of Dix and Hallpike to multidisciplinary reviews published in 2026. Together they trace the full arc of vestibular medicine: from histological observation, through clinical standardization, to the recognition that even well-established diagnostic protocols fail under real-world conditions. Figure 2 illustrates this chronological progression and the key contributions of each milestone work.

The diagnostic framework underpinning modern vestibular medicine was established by Dix and Hallpike in 1952, who formally distinguished Meniere’s disease, vestibular neuronitis, and benign paroxysmal positional nystagmus through clinicopathological correlation and introduced the positional maneuver that carries their name [5]. This foundational classification required 65 years to be codified into an evidence-based clinical guideline; the 2017 American Academy of Otolaryngology update synthesized 27 randomized controlled trials and 20 systematic reviews to issue strong recommendations for the Dix-Hallpike maneuver as the diagnostic gold standard for posterior canal BPPV, the Epley maneuver as first-line treatment, and explicit recommendations against vestibular suppressants and routine neuroimaging [7]. The GRACE-3 guidelines published in 2023 extended this framework to emergency department settings, rejecting the traditional symptom-quality paradigm in favor of a timing-and-triggers approach and categorizing patients into three clinical syndromes (AVS, s-EVS, t-EVS) each demanding a distinct diagnostic pathway [8]. The thread connecting all three works is consistent: bedside physical examination specifically eye movement assessment via HINTS and the Dix-Hallpike maneuver is both underutilized and undervalued relative to neuroimaging, and clinician training rather than technology is identified as the primary barrier to better outcomes.

A recurring tension across the pharmacological papers is the tradeoff between short-term symptom relief and long-term recovery. Dimenhydrinate, an H1-receptor antagonist widely prescribed for acute vertigo, is shown to produce significantly lower rates of objective vestibular ocular reflex recovery at three months compared to non-medicated controls, directly conflicting with the 2017 guideline’s recommendation against vestibular suppressants for BPPV [7]. The combined steroid and isosorbide trial for acute low-tone sensorineural hearing loss demonstrates a parallel tradeoff: combined therapy achieves higher absolute hearing gains at 125–500 Hz but fails to reach statistical significance on a 30-patient sample, leaving the benefit-risk calculation unresolved. The saccadic oscillation case series adds a third dimension, showing that cholinesterase inhibitors used for Alzheimer’s management can themselves induce dizziness through GABAergic suppression in the superior colliculus, resolving only upon dose reduction or discontinuation. Across these three papers, the common clinical implication is that pharmacological management of vestibular symptoms requires individualized risk stratification rather than protocol-driven prescription.

Vestibular migraine (VM) and central positional nystagmus (CPN) are intrinsically linked to otoconial disease, specifically benign paroxysmal positional vertigo (BPPV), as both represent primary differential diagnoses that frequently mimic the temporal characteristics of BPPV [61]. Research indicates a strong epidemiologic association, where migraine is three times more common in patients with idiopathic BPPV, potentially due to vascular damage to the labyrinth or genetic factors that predispose migraineurs to otoconial displacement [62,63]. While VM and paroxysmal CPN can present as purely positional vertigo, they are distinguished from BPPV by nystagmus patterns that are typically central such as pure vertical or torsional movements rather than being aligned with a specific semicircular canal. This clinical information is limited but invaluable for modern AI, which can leverage the “explainable AI” and mathematical ocular motor models described in the sources to automate the differentiation between central and peripheral vestibular pathologies.

Even when clinical guidelines are clear, correct diagnosis at the diagnostic boundaries remains inconsistent. The retrospective series of 273 emergency department patients initially diagnosed with vestibular neuritis demonstrates that approximately 20.5% actually had lateral semicircular canal BPPV presenting with pseudo-spontaneous nystagmus, a gravity-sensitive, direction-changing pattern indistinguishable from true spontaneous nystagmus without the Head Pitch Test [43]. The Meniere’s variant case report further illustrates that existing diagnostic criteria, which require low-to-medium frequency hearing loss, may exclude clinically identical presentations with isolated high-frequency involvement, suggesting a diagnostic gap for conditions such as HIVES. The interobserver agreement study provides the most direct quantification of this ambiguity: six vestibular medicine specialists reviewing 240 eye movement recordings achieved only fair-to-moderate consensus (kappa 0.40), with agreement dropping further for rare canal variants [46]. The reposition chair study completes the picture, showing that even after diagnosis is established, 31 patients who had failed standard manual repositioning required mechanical intervention to achieve remission, with DHI scores halving and HADS anxiety scores improving significantly underscoring that diagnostic failure and treatment failure are linked and that current clinical pathways leave a measurable patient population unserved [41]. Recent studies [64,65,66] in healthy controls have shown that low-velocity spontaneous or positional nystagmus can be frequent, especially when fixation is removed and sensitive video-oculographic systems are used. Therefore, future AI or telemedicine tools should not only detect nystagmus, but also quantify its slow-phase velocity, direction, temporal profile, paroxysmal nature, reproducibility, and association with symptoms.

### 3.3. Hardware Landscape Analysis: Cost, Fixation Suppression, and Platform Gaps

The smartphone and hardware papers collectively map a cost-accessibility spectrum that ranges from commercially available infrared cameras costing USD 25 and 3D-printed goggle frames at USD 13, to Apple ARKit-based applications on consumer smartphones, up to dedicated augmented reality headsets. Across 12 papers, the shared clinical objective is extending objective eye movement recording beyond the subspecialty clinic to emergency departments, primary care offices, and patients’ homes. Table 2 summarizes and compares the key technical specifications of each system reviewed.

The reviewed hardware spans four orders of magnitude in cost, from 3D-printed goggle frames produced for approximately USD 13 in filament [59] and wearable strabismus screening devices at GBP 40 [50], through consumer smartphones already owned by patients, up to augmented reality headsets at moderate institutional cost and gold-standard IR VOG systems reaching USD 40,000 [9]. Crucially, cost reduction does not map linearly onto accuracy loss: the EyePhone application on a standard iPhone achieves *r* = 0.98 horizontal SPV correlation with VOG [52], and the 3D-printed SVFD is described as superior to traditional VNG for torsional component visualization in some gaze positions [55]. The Raspberry Pi-based PSCN achieves 80% accuracy against VNG at a fraction of the cost [53], while the VertiScope smartphone system produces mean SPV measurements statistically indistinguishable from gold-standard VNG across a 39-participant cohort [54]. The practical implication is that the cost barrier to objective nystagmus assessment has largely been dissolved at the hardware level; the remaining barrier is validation breadth and regulatory acceptance.

The single technical limitation shared across the majority of smartphone-based systems is the inability to remove visual fixation during testing. Fixation suppression is physiologically essential because it allows patients to compensate for vestibular-induced nystagmus, reducing or eliminating the signal being measured. The original Android app by van Bonn et al. explicitly attributes its 15% directional sensitivity in caloric trials to fixation contamination [11]. ARKit-based systems including Parker et al. and the EyePhone studies operate in ambient light without occlusion, limiting their utility for caloric and spontaneous nystagmus assessment [10,52]. In contrast, goggle-based solutions the cardboard iCapNYS goggles [58], the 3D-printed home monitoring system [59], the SVFD [55], the Raspberry Pi PSCN [53], and the AR smart glasses [56] all provide fixation removal as a design requirement, making them more suitable for spontaneous and positional nystagmus assessment. The hardware taxonomy therefore divides into two functional classes: fixation-present smartphone trackers best suited for optokinetic and quantified gaze testing, and fixation-removing wearable systems suited for spontaneous, positional, and caloric assessment.

Beyond fixation handling, the reviewed systems diverge across several technical parameters that govern data quality and deployment feasibility. Sampling rate is a primary determinant of waveform fidelity: most platforms operate at 30 Hz [11,12] or 60 Hz [10,51,52], and the aEYE system demonstrates that detection performance degrades gracefully as frame rate drops toward 15 Hz, a finding directly relevant to bandwidth-constrained telemedicine [57]. Calibration requirements vary substantially and increasingly trend toward calibration-free operation: ConVNG produces VOG-equivalent slow-phase velocity from standard RGB video without formal calibration by relying on anthropomorphic assumptions [12], while OpenNystagmus achieves the lowest calibration error in the reviewed corpus (0.38°, below the 1° clinical threshold) without a dedicated calibration protocol [22], indicating that the historical need for per-session calibration is no longer a hard constraint for accessible deployment. Head stabilization remains a design trade-off between accuracy and accessibility, with some systems incorporating dedicated fixtures such as the VertiScope PrecisionView Stand and chin rest to minimize motion artifacts [54], whereas others are explicitly engineered for hands-free, distance-agnostic use [22]. Finally, the locus of processing distinguishes edge from cloud architectures: on-device edge computing, as implemented in the visible-light pipeline of Su et al., reduces latency and preserves user privacy in low-resource settings [49], in contrast to cloud-based pipelines that centralize computation at the cost of connectivity dependence.

Three independent validation studies, Parker et al. [10], Phillips et al. [51], and Bastani et al. [52], all deploy Apple’s ARKit framework and report high-fidelity eye and head tracking at 60 FPS using the iPhone’s infrared dot projector and front-facing camera. This convergence makes ARKit the leading candidate platform validated platform for smartphone-based quantitative oculometry, with *r >* 0.94 correlation to VOG across multiple study designs. However, this creates a platform asymmetry: Android devices, which constitute the majority of the global smartphone market and dominate in low-and middle-income countries where equitable access is most needed, lack equivalent validated frameworks.

The OpenCV-based Android app by van Bonn et al. [11] and the CHT-based visible-light algorithm by Su et al. [49] represent early steps toward platform-agnostic solutions, but neither has achieved the measurement fidelity of ARKit implementations. Closing this gap is the most consequential near-term hardware challenge for democratizing vestibular diagnostics globally.

### 3.4. Telemedicine Analysis: Episodic Capture and Remote Diagnostic Frameworks

**Figure 3:**
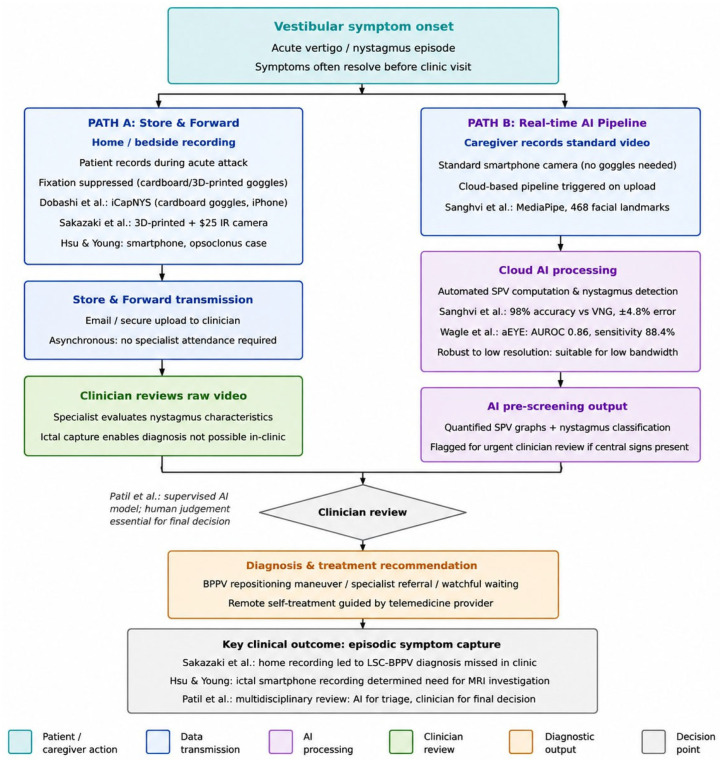
Patient-centered telemedicine pipeline for vestibular diagnostics. Papers reviewed map onto distinct stages of the pipeline: symptom onset and home recording (Dobashi et al., Sakazaki et al.), transmission and cloud processing (Sanghvi et al. [14]), AI-assisted pre-screening (Wagle et al. [57]), and clinician review and diagnosis (Patil et al. [60], Hsu et al. [40]). Arrows indicate data flow direction.

The published telemedicine articles converge on a single clinical problem that no in-clinic technology can solve vestibular symptoms are episodic, and patients are almost always asymptomatic by the time they reach a specialist. The five papers reviewed represent four distinct architectural responses to this challenge, ranging from patient-initiated home recording to cloud-based AI pipelines, as illustrated in Figure 3.

The fundamental architectural choice in telemedicine for vestibular disorders is between store-and-forward models where the patient records during an attack and transmits for later review and real-time pipelines where AI processes video immediately and delivers a quantified output for synchronous clinician assessment. The iCapNYS system exemplifies the store-and-forward approach, using recyclable cardboard goggles to record spontaneous nystagmus during a Meniere’s attack and emailing the recording directly to a clinician, with SPV measurements of 12.0°/s closely matching the 12.6°/s obtained by gold-standard Frenzel glasses [58]. The 3D-printed home monitoring system extends this further: a patient with no clinical findings on prior standard testing successfully captured geotropic direction-changing nystagmus at home using a USD 25 infrared camera, leading directly to a definitive BPPV diagnosis confirmed by Gufoni maneuver response [59]. In contrast, the Sanghvi et al. cloud-based AI framework processes uploaded smartphone video in near real-time, computing SPV automatically and generating visual graphs for clinician review with 98% accuracy against VNG and error margin of only ±4.8% [14]. The opsoclonus case by Hsu et al. represents the most clinically urgent scenario, where smartphone recording during an active attack provided the only objective evidence of 15 Hz multi-directional eye movements, directly informing the decision to investigate for viral encephalitis, an outcome impossible without ictal capture [40].

The five telemedicine papers diverge on where AI authority ends and clinician judgment begins, reflecting an unresolved design question with direct implications for regulatory approval and patient safety. The Sanghvi et al. framework is explicitly physician-focused: data collection and SPV computation are automated, but final diagnostic interpretation and management recommendations are reserved for the clinician, with the AI functioning as a quantification tool rather than a diagnostic agent [14]. The aEYE system by Wagle et al. takes a different position, targeting non-expert detection of nystagmus in resource-limited settings where a specialist is unavailable, achieving AUROC 0.86 and sensitivity 88.4% on real-world AVERT trial data while maintaining performance at reduced frame rates and resolutions suited to telemedicine bandwidth constraints [57]. The multidisciplinary review by Patil et al. contextualizes this tension at the field level, arguing that LLMs and computer vision tools are best positioned for initial triage rather than definitive diagnosis, and that successful clinical deployment requires ethical frameworks for data privacy alongside rigorous real-world validation [60]. The consensus emerging across these papers is a tiered model: patient-initiated ictal recording, followed by automated AI pre-screening and quantification, followed by asynchronous specialist review, a pipeline that preserves clinical authority while solving the fundamental problem of symptom capture.

## 4. Conclusions

This review documents a vestibular medicine field in rapid transition: from specialist-bound, hardware-intensive assessment toward a distributed, AI-assisted diagnostic ecosystem that is increasingly accessible at the point of care. Across 50 papers spanning 1952 to 2026, what stands out most is how unevenly progress is distributed: the technical building blocks are far more evolved than the clinical and regulatory scaffolding needed to bring them into routine practice.

The capacity to capture objective eye-movement data outside the specialist clinic is now well established. Systems ranging from USD 13 3D-printed goggles to ARKit-based smartphone applications demonstrate slow-phase velocity measurement comparable to video-oculography under the conditions tested, and on the analytical side, machine learning and deep learning models consistently outperform established clinical scores for central–peripheral differentiation. The most decisive finding here is that head-position data is not supplementary but essential: its removal collapses multimodal BPPV classification accuracy by over 40 percentage points, with direct implications for the performance ceiling of nystagmus-only frameworks, where the evidence is in the step from controlled demonstration to a clinically proven tool. Most reported results derive from single-site, retrospective, or proof-of-concept studies, and prospective, multi-site evaluation against adjudicated clinical reference standards remains the exception rather than the rule. The path toward regulated diagnostic use is still in early stages, constrained by the absence of explainable AI mechanisms sufficient to support approval and by dataset scarcity for rare canal subtypes. Even where a system performs well, routine deployment is held back by practical obstacles that performance alone does not resolve, such as the absence of visual fixation suppression in most smartphone platforms, the lack of validated frameworks on the Android devices that dominate low- and middle-income markets, and unresolved questions of data privacy and clinical oversight.

Across all domains, the episodic-capture problem, the fundamental reason dangerous vestibular diagnoses are so frequently missed, is beginning to be addressed by patient-initiated ictal recording systems that enable store-and-forward telemedicine without specialist attendance, as illustrated by case reports in which home recordings contributed to diagnoses that prior clinic visits had failed to capture. Yet the same evidence underscores that these systems are best positioned to augment, not replace, clinical judgment, and that the question of how much diagnostic authority can be delegated to automated systems before a clinician must be in the loop remains genuinely open. The barriers that remain are therefore not primarily ones of technical capability but of validation breadth, interpretability, regulatory acceptance, and equitable deployment. Whether the diagnostic access gap in vestibular medicine is ultimately closed or merely narrowed will depend on resolving these through multimodal data fusion, federated dataset development, and rigorous prospective, real-world trials.

## Figures and Tables

**Table 1:** Performance summary of AI-based nystagmus and vertigo diagnostic systems reviewed. Category: ML = Machine Learning, DL = Deep Learning, LLM = Large Language/Vision Model. *n* refers to patients for ML studies and videos or clips for DL studies. Systematic reviews (Chaturvedi et al. [15]; Dai et al. [21]) are excluded as they report no primary performance data. Dashes indicate metric not applicable or not reported.

Reference	Year	Cat.	Method	Task	Sensing Modality	Hardware/Platform	Dataset Type	*n*	Metric	Value
Lai et al. [16]	2026	ML	RF + AdaBoost	Vertigo triage (central vs. peripheral)	VOG + vHIT features	—	Patient records (2 institutions)	294 patients	Accuracy/AUC	97%/0.95
Ahmadi et al. [13]	2020	ML	MultiGMC, RF, ANN	Central vs. peripheral AVS	Quantitative VOG + MRI + neuro-otologic exam	—	Multimodal patient dataset	108 patients	AUC	0.96
Zhao et al. [17]	2025	ML	CCA + GBDT	Multi-class vertigo (16 categories)	Bedside ocular-motor exam + history	Video goggles	Retrospective multimodal records	1003 patients	Accuracy/F1	98.11%/95.43%
Newman et al. [18]	2019	ML	SVM + LDA + Boosted trees (ensemble)	Ambulatory nystagmus detection	Electronystagmography (ENG)	CAVA wearable device	30-day ambulatory recordings	17 patients	Accuracy	98.77%
Yang et al. [19]	2025	ML	CatBoost (SPV profiles)	BPV vs. mimics	VNG	—	VNG recordings	476 VNG recordings	Accuracy	93.3%
Anh et al. [20]	2022	ML	5-algorithm ensemble	PV vs. non-PV classification	Caloric + stabilometry + eye-tracking	—	Multi-feature patient dataset	1009 patients	Accuracy (consensus)	85%
Friedrich et al. [12]	2023	DL	CNN (ConVNG, 17 landmarks)	SPV quantification (OKN)	RGB video (30 Hz)	Standard smartphone	Prospective + retrospective clinical video	—	VOG deviation	≤5%
Phillips [22]	2025	DL	Haar + GroundingDINO + EllSeg	OKN quantification (marker-free)	Smartphone video	Smartphone (hands-free)	Smartphone OKN video	—	Calibration error	0.38°
Mun et al. [23]	2024	DL	2D U-Net + CNN1D	BPPV nystagmus detection	Infrared VOG	—	Infrared video clips	3300+ video clips	Accuracy/F1	91.02%/92.68%
Cho et al. [24]	2024	DL	Tri-class ocular segmentation	Real-time nystagmus tracking	Clinical video + synthetic VR	Workstation/consumer GPU	Clinical + synthetic images	82,000+ images	mIoU/Speed	0.97/194 FPS
Zhang et al. [25]	2021	DL	TBSIN + dense optical flow	Torsional nystagmus (pc-BPPV)	VNG	—	VNG video	—	Frame acc./F1	85.73%/81.00%
Kong et al. [26]	2023	DL	LiteFlowNet + NVCN (ConvNeXt + LSTM)	BPPV pattern classification	Infrared video	—	Infrared videos	728 videos	Accuracy (torsional)	94.91% (97.75%)
Lu et al. [27]	2024	DL	BKTDN + cross-attention (multimodal)	Multimodal BPPV diagnosis	Eye video + 3D head position	—	Eye-movement video + head position	518 patients	Accuracy	81.7%
Lee et al. [28]	2023	DL	U-Net + ellipse-fitting (ANyEye)	BPPV nystagmus extraction	VNG (dark-field)	—	VNG recordings	—	Detection rate	91.26%
Lin et al. [29]	2026	DL	SAM + 1D CNN (zero-shot)	Horizontal nystagmus classification	—	—	LPW benchmark + clinical video	—	Precision	81%
Liu et al. [30]	2025	DL	Egeunet + FFT + SIFT	BPPV signal diagnosis	Wearable video capture	Wearable remote-clinic device	Eye-region video	—	Accuracy	93.33%
Azhari et al. [31]	2025	DL	YOLOv11s + LSTM	BPPV nystagmus classification	VNG	—	VNG recordings	2500 VNG recordings	Accuracy	82%
Lim et al. [32]	2019	DL	2D CNN (spatiotemporal grid)	BPPV canal identification	Video goggles	Video goggles	Annotated video clips (3467 pts)	91,778 video clips	Sensitivity/Specificity	>87%/>87%
Abulail et al. [33]	2025	DL	MediaPipe (468 landmarks) + OpenCV	Facial movement disorder detection	RGB webcam	Laptop camera/Streamlit	Video + static images	—	Accuracy	93–95%
Noda et al. [35]	2025	LLM	GPT-4V (zero-shot, CoT, MR prompting)	Nystagmus pattern classification	Pupil-trace images/CSV	GPT-4V API	Trace images + coordinate data	—	Accuracy	17–38%
Mikhail et al. [36]	2026	LLM	Gemini 2.0 (zero-shot)	Ocular motility disorder diagnosis	RGB clinical video	Gemini 2.0 API	Real-world clinical videos	129 videos	Overall/Best condition	37.7%/81.1%
Lacinski et al. [34]	2024	LLM	Survey of LLMs (ChatDoctor, Almanac)	AI for space medicine (vestibular)	—	—	119 prioritized conditions	119 conditions	—	—

**Table 2:** Comparison of smartphone and portable hardware systems reviewed. Cost estimates are approximate and reflect hardware only. FPS = frames per second. VOG = video-oculography. VNG = videonystagmography. ARKit = Apple ARKit framework. IR = infrared. CV = computer vision.

Reference	Year	Device/Platform	Approx. Cost	FPS	Fixation Removal	Validated Against	Setting
van Bonn et al. [11]	2022	Android app (OpenCV)	Smartphone only	30	No	VNG goggles	Emergency/Telemedicine
Bastani et al. [9]	2024	Smartphone vs. IR goggles (review)	USD 0–40,000	Varies	Partial	IR VOG goggles	Subspecialty clinic
Parker et al. [10]	2022	iPhone (ARKit)	Smartphone only	60	No	VOG goggles	Telemedicine/Research
Hartness et al. [48]	2025	Various (review/meta-analysis)	Varies	Varies	Varies	APCT (prism cover)	Ophthalmology clinic
Su et al. [49]	2025	iPhone 12 (visible light)	Smartphone only	30	No	Ground truth coords	Telemedicine/Screening
Zhao et al. [50]	2025	3D-printed wearable + IR cams	∼GBP 40	—	Yes (goggle)	PCT (prism cover)	School/Community screening
Phillips et al. [51]	2025	iPhone (ARKit, 1080p)	Smartphone only	60	No	Expert clinician review	ED/Telemedicine
Bastani et al. [52]	2024	iPhone (ARKit)	Smartphone only	60	No	VOG goggles	ED/Telemedicine
Sefein et al. [53]	2019	Raspberry Pi + goggle (Bluetooth)	Low cost	—	Yes (goggle)	VNG	Rural clinic
Sakharkar et al. [54]	2025	Smartphone + PrecisionView Stand	Smartphone only	—	Partial	VNG	Clinical/Outpatient
Özel [55]	2026	3D-printed SVFD + smartphone	Low cost	—	Yes (goggle)	VNG	Clinic/Telemedicine
Wu et al. [56]	2025	J7EF Gaze AR smart glasses	Moderate	—	Yes (headset)	VNG Ulmer	ED/Hospital

## Data Availability

No new data were created or analyzed in this study. Data sharing is not applicable to this article.
